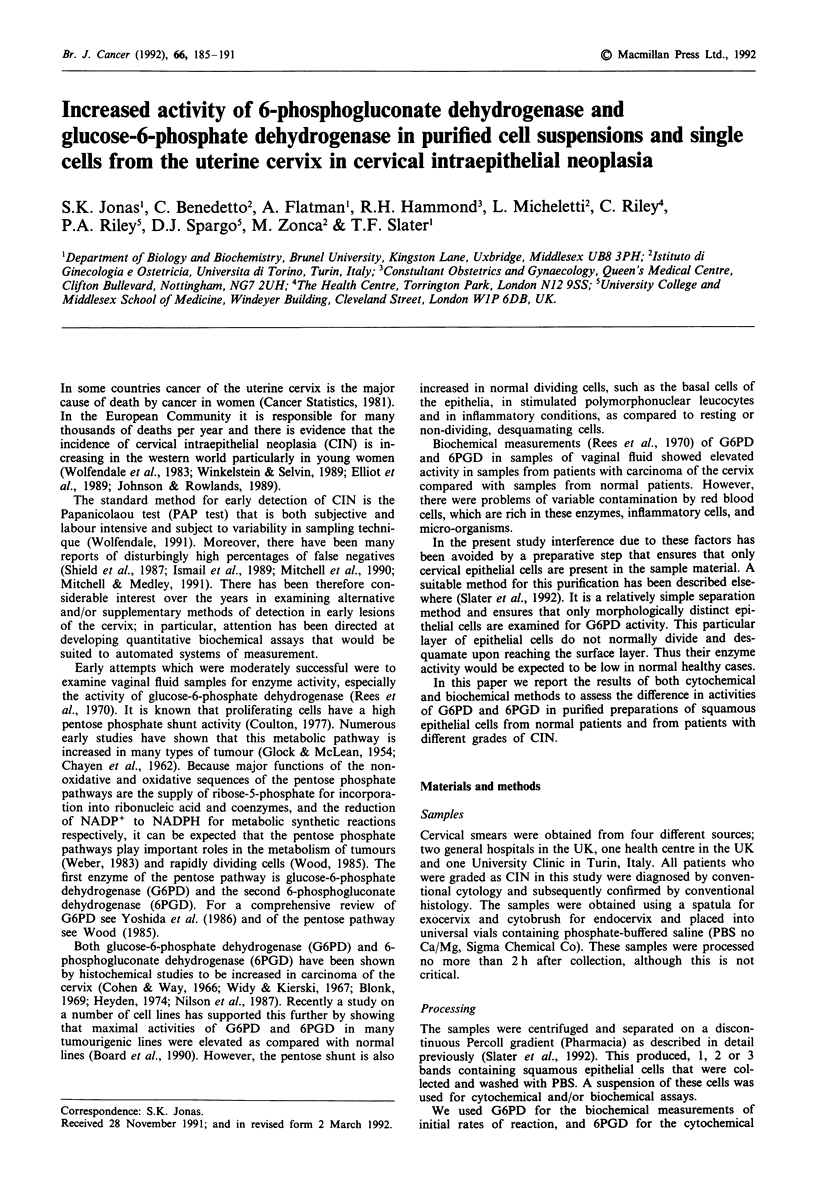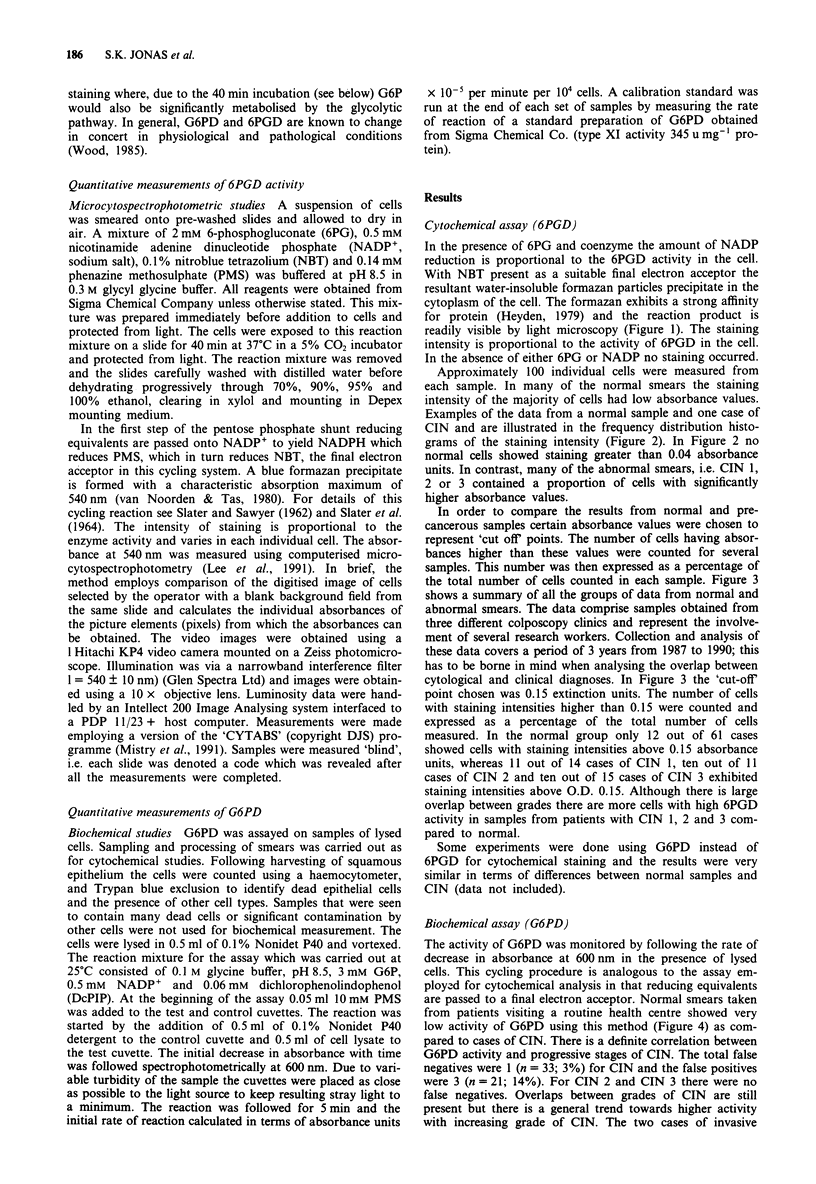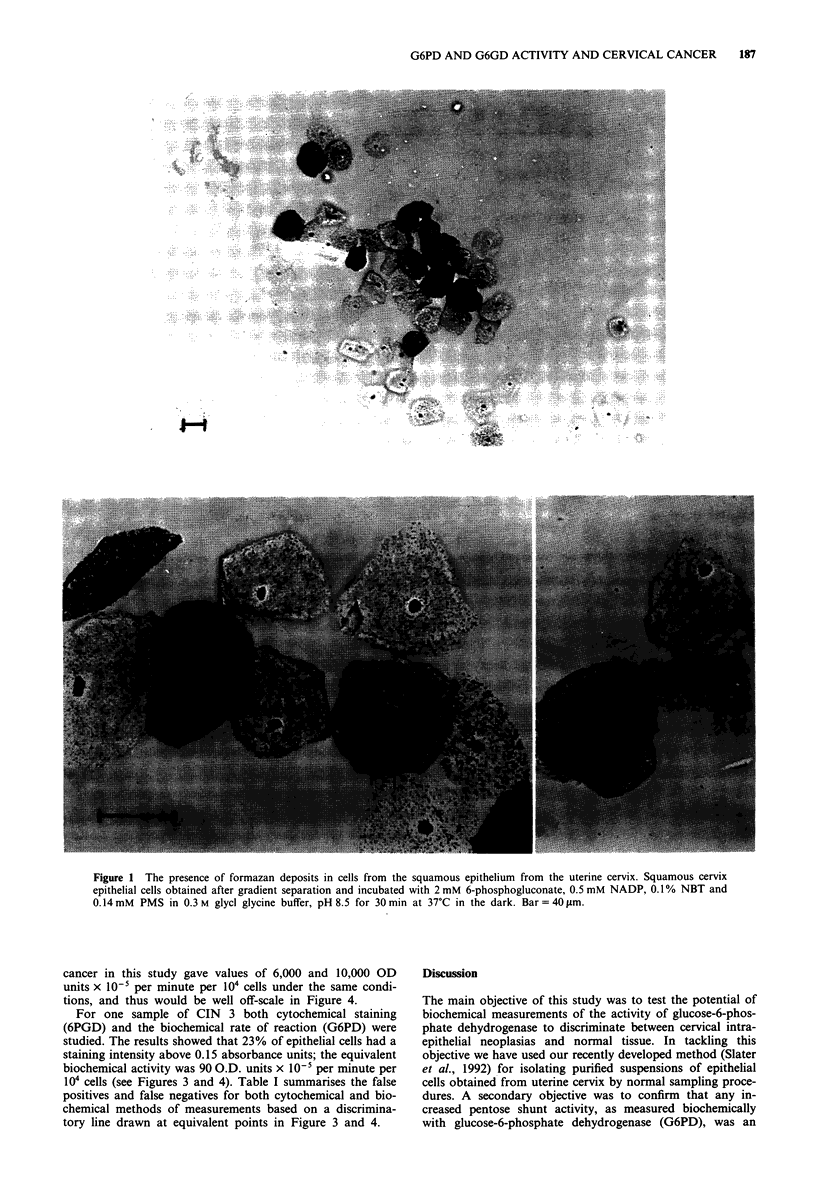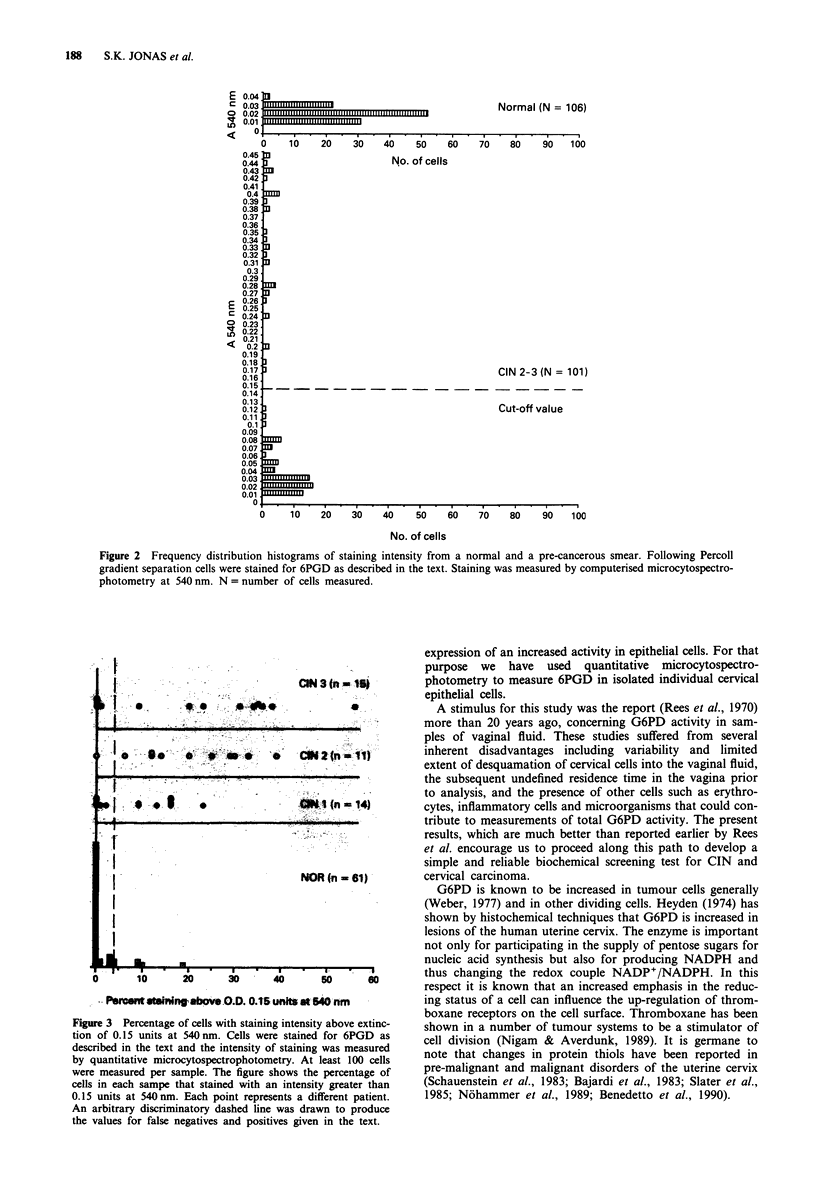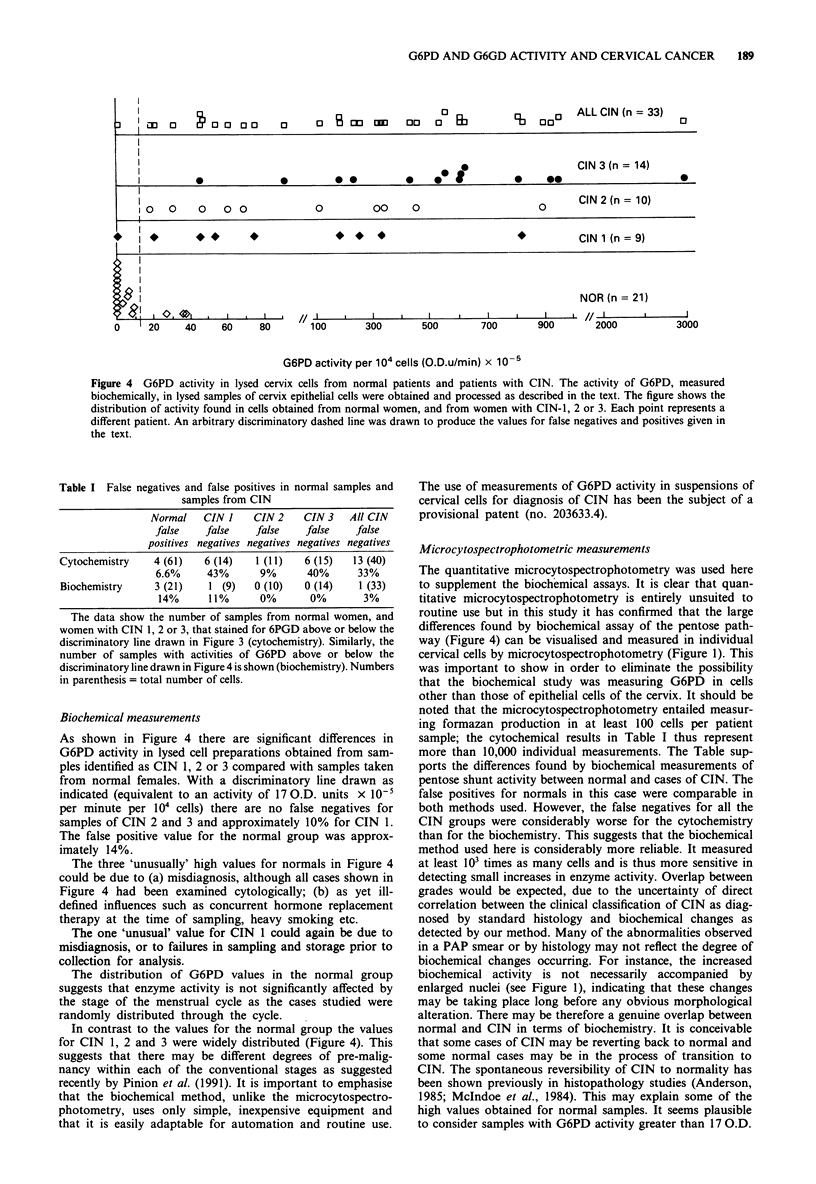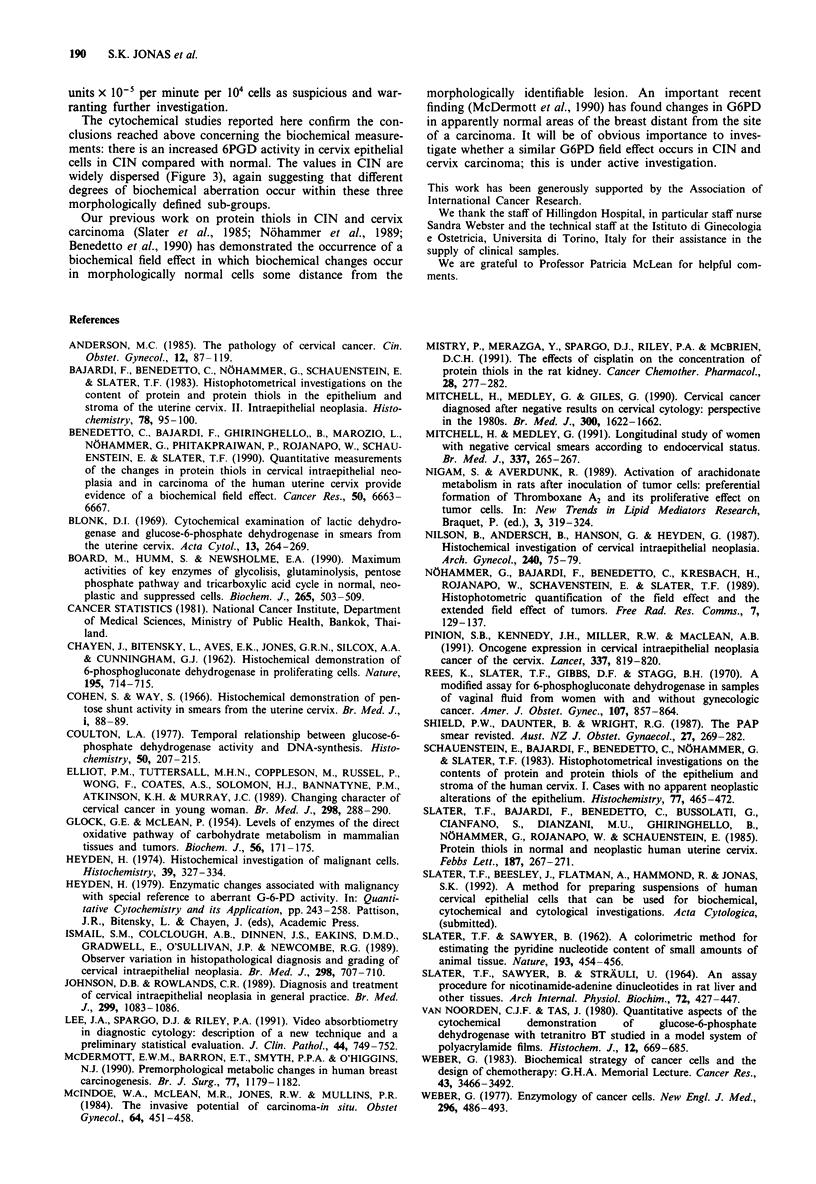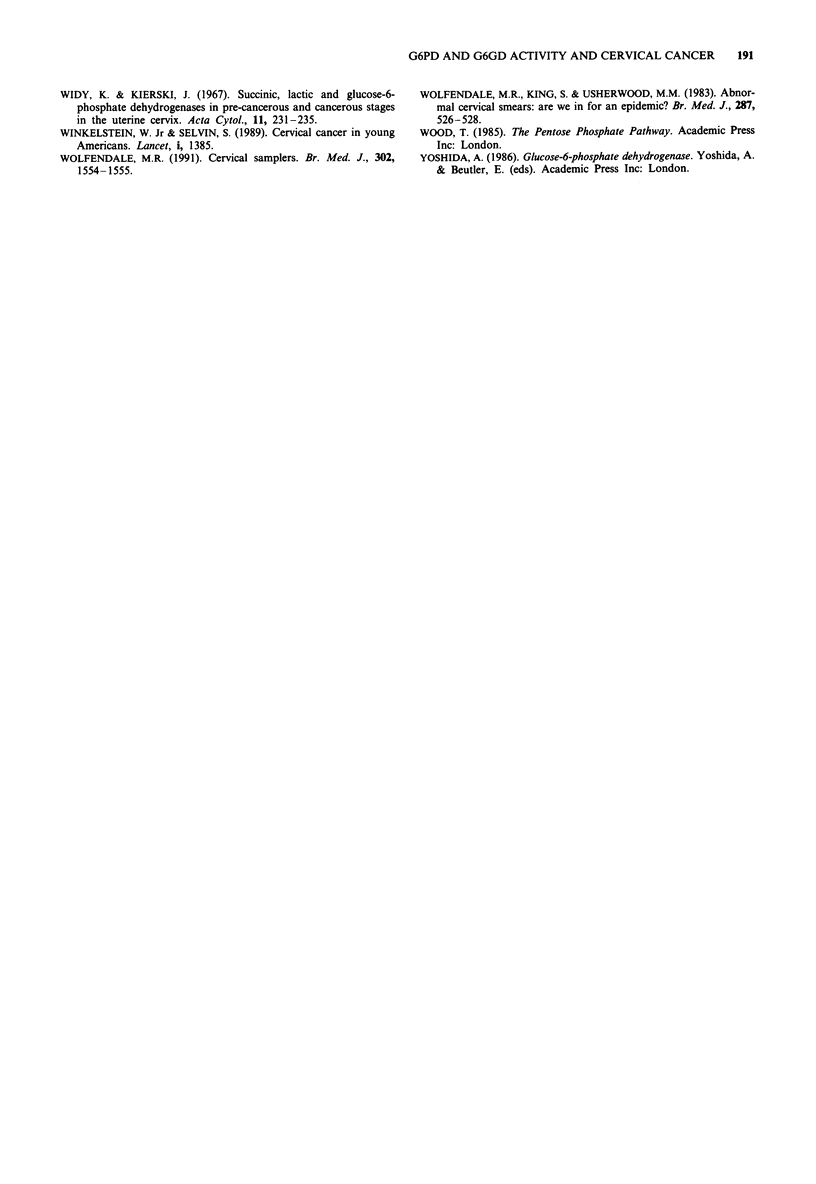# Increased activity of 6-phosphogluconate dehydrogenase and glucose-6-phosphate dehydrogenase in purified cell suspensions and single cells from the uterine cervix in cervical intraepithelial neoplasia.

**DOI:** 10.1038/bjc.1992.240

**Published:** 1992-07

**Authors:** S. K. Jonas, C. Benedetto, A. Flatman, R. H. Hammond, L. Micheletti, C. Riley, P. A. Riley, D. J. Spargo, M. Zonca, T. F. Slater

**Affiliations:** Department of Biology and Biochemistry, Brunel University, Uxbridge, Middlesex, UK.

## Abstract

**Images:**


Br. J. Cancer (1992), 66, 185–191 © Macmillan Press Ltd., 1992

### Increased activity of 6-phosphogluconate dehydrogenase and glucose-6-phosphate dehydrogenase in purified cell suspensions and single cells from the uterine cervix in cervical intraepithelial neoplasia

S.K. Jonas[1], C. Benedetto[2], A. Flatman[1], R.H. Hammond[3], L. Micheletti[2], C. Riley[4], P.A. Riley[5], D.J. Spargo[5], M. Zonca[2] & T.F. Slater[1]

[1]*Department of Biology and Biochemistry, Brunel University, Kingston Lane, Uxbridge, Middlesex UB8 3PH;* [2]*Istituto di Ginecologia e Ostetricia, Universita di Torino, Turin, Italy;* [3]*Constultant Obstetrics and Gynaecology, Queen's Medical Centre, Clifton Bullevard, Nottingham, NG7 2UH;* [4]*The Health Centre, Torrington Park, London N12 9SS;* [5]*University College and Middlesex School of Medicine, Windeyer Building, Cleveland Street, London W1P 6DB, UK.*

In some countries cancer of the uterine cervix is the major cause of death by cancer in women (Cancer Statistics, 1981). In the European Community it is responsible for many thousands of deaths per year and there is evidence that the incidence of cervical intraepithelial neoplasia (CIN) is increasing in the western world particularly in young women (Wolfendale *et al.*, 1983; Winkelstein & Selvin, 1989; Elliot *et al.*, 1989; Johnson & Rowlands, 1989).

The standard method for early detection of CIN is the Papanicolaou test (PAP test) that is both subjective and labour intensive and subject to variability in sampling technique (Wolfendale, 1991). Moreover, there have been many reports of disturbingly high percentages of false negatives (Shield *et al.*, 1987; Ismail *et al.*, 1989; Mitchell *et al.*, 1990; Mitchell & Medley, 1991). There has been therefore considerable interest over the years in examining alternative and/or supplementary methods of detection in early lesions of the cervix; in particular, attention has been directed at developing quantitative biochemical assays that would be suited to automated systems of measurement.

Early attempts which were moderately successful were to examine vaginal fluid samples for enzyme activity, especially the activity of glucose-6-phosphate dehydrogenase (Rees *et al.*, 1970). It is known that proliferating cells have a high pentose phosphate shunt activity (Coulton, 1977). Numerous early studies have shown that this metabolic pathway is increased in many types of tumour (Glock & McLean, 1954; Chayen *et al.*, 1962). Because major functions of the non-oxidative and oxidative sequences of the pentose phosphate pathways are the supply of ribose-5-phosphate for incorporation into ribonucleic acid and coenzymes, and the reduction of $NADP^+$ to NADPH for metabolic synthetic reactions respectively, it can be expected that the pentose phosphate pathways play important roles in the metabolism of tumours (Weber, 1983) and rapidly dividing cells (Wood, 1985). The first enzyme of the pentose pathway is glucose-6-phosphate dehydrogenase (G6PD) and the second 6-phosphogluconate dehydrogenase (6PGD). For a comprehensive review of G6PD see Yoshida *et al.* (1986) and of the pentose pathway see Wood (1985).

Both glucose-6-phosphate dehydrogenase (G6PD) and 6-phosphogluconate dehydrogenase (6PGD) have been shown by histochemical studies to be increased in carcinoma of the cervix (Cohen & Way, 1966; Widy & Kierski, 1967; Blonk, 1969; Heyden, 1974; Nilson *et al.*, 1987). Recently a study on a number of cell lines has supported this further by showing that maximal activities of G6PD and 6PGD in many tumourigenic lines were elevated as compared with normal lines (Board *et al.*, 1990). However, the pentose shunt is also increased in normal dividing cells, such as the basal cells of the epithelia, in stimulated polymorphonuclear leucocytes and in inflammatory conditions, as compared to resting or non-dividing, desquamating cells.

Biochemical measurements (Rees *et al.*, 1970) of G6PD and 6PGD in samples of vaginal fluid showed elevated activity in samples from patients with carcinoma of the cervix compared with samples from normal patients. However, there were problems of variable contamination by red blood cells, which are rich in these enzymes, inflammatory cells, and micro-organisms.

In the present study interference due to these factors has been avoided by a preparative step that ensures that only cervical epithelial cells are present in the sample material. A suitable method for this purification has been described elsewhere (Slater *et al.*, 1992). It is a relatively simple separation method and ensures that only morphologically distinct epithelial cells are examined for G6PD activity. This particular layer of epithelial cells do not normally divide and desquamate upon reaching the surface layer. Thus their enzyme activity would be expected to be low in normal healthy cases.

In this paper we report the results of both cytochemical and biochemical methods to assess the difference in activities of G6PD and 6PGD in purified preparations of squamous epithelial cells from normal patients and from patients with different grades of CIN.

### Materials and methods

### *Samples*

Cervical smears were obtained from four different sources; two general hospitals in the UK, one health centre in the UK and one University Clinic in Turin, Italy. All patients who were graded as CIN in this study were diagnosed by conventional cytology and subsequently confirmed by conventional histology. The samples were obtained using a spatula for exocervix and cytobrush for endocervix and placed into universal vials containing phosphate-buffered saline (PBS no Ca/Mg, Sigma Chemical Co). These samples were processed no more than 2 h after collection, although this is not critical.

### *Processing*

The samples were centrifuged and separated on a discontinuous Percoll gradient (Pharmacia) as described in detail previously (Slater *et al.*, 1992). This produced, 1, 2 or 3 bands containing squamous epithelial cells that were collected and washed with PBS. A suspension of these cells was used for cytochemical and/or biochemical assays.

We used G6PD for the biochemical measurements of initial rates of reaction, and 6PGD for the cytochemical

Correspondence: S.K. Jonas.
Received 28 November 1991; and in revised form 2 March 1992.

staining where, due to the 40 min incubation (see below) G6P would also be significantly metabolised by the glycolytic pathway. In general, G6PD and 6PGD are known to change in concert in physiological and pathological conditions (Wood, 1985).

*Quantitative measurements of 6PGD activity*

*Microcytospectrophotometric studies* A suspension of cells was smeared onto pre-washed slides and allowed to dry in air. A mixture of 2 mM 6-phosphogluconate (6PG), 0.5 mM nicotinamide adenine dinucleotide phosphate ($NADP^+$, sodium salt), 0.1% nitroblue tetrazolium (NBT) and 0.14 mM phenazine methosulphate (PMS) was buffered at pH 8.5 in 0.3 M glycyl glycine buffer. All reagents were obtained from Sigma Chemical Company unless otherwise stated. This mixture was prepared immediately before addition to cells and protected from light. The cells were exposed to this reaction mixture on a slide for 40 min at 37°C in a 5% $CO_2$ incubator and protected from light. The reaction mixture was removed and the slides carefully washed with distilled water before dehydrating progressively through 70%, 90%, 95% and 100% ethanol, clearing in xylol and mounting in Depex mounting medium.

In the first step of the pentose phosphate shunt reducing equivalents are passed onto $NADP^+$ to yield NADPH which reduces PMS, which in turn reduces NBT, the final electron acceptor in this cycling system. A blue formazan precipitate is formed with a characteristic absorption maximum of 540 nm (van Noorden & Tas, 1980). For details of this cycling reaction see Slater and Sawyer (1962) and Slater *et al.* (1964). The intensity of staining is proportional to the enzyme activity and varies in each individual cell. The absorbance at 540 nm was measured using computerised microcytospectrophotometry (Lee *et al.*, 1991). In brief, the method employs comparison of the digitised image of cells selected by the operator with a blank background field from the same slide and calculates the individual absorbances of the picture elements (pixels) from which the absorbances can be obtained. The video images were obtained using a l Hitachi KP4 video camera mounted on a Zeiss photomicroscope. Illumination was via a narrowband interference filter l = 540 ± 10 nm) (Glen Spectra Ltd) and images were obtained using a 10 × objective lens. Luminosity data were handled by an Intellect 200 Image Analysing system interfaced to a PDP 11/23 + host computer. Measurements were made employing a version of the 'CYTABS' (copyright DJS) programme (Mistry *et al.*, 1991). Samples were measured 'blind', i.e. each slide was denoted a code which was revealed after all the measurements were completed.

*Quantitative measurements of G6PD*

*Biochemical studies* G6PD was assayed on samples of lysed cells. Sampling and processing of smears was carried out as for cytochemical studies. Following harvesting of squamous epithelium the cells were counted using a haemocytometer, and Trypan blue exclusion to identify dead epithelial cells and the presence of other cell types. Samples that were seen to contain many dead cells or significant contamination by other cells were not used for biochemical measurement. The cells were lysed in 0.5 ml of 0.1% Nonidet P40 and vortexed. The reaction mixture for the assay which was carried out at 25°C consisted of 0.1 M glycine buffer, pH 8.5, 3 mM G6P, 0.5 mM $NADP^+$ and 0.06 mM dichlorophenolindophenol (DcPIP). At the beginning of the assay 0.05 ml 10 mM PMS was added to the test and control cuvettes. The reaction was started by the addition of 0.5 ml of 0.1% Nonidet P40 detergent to the control cuvette and 0.5 ml of cell lysate to the test cuvette. The initial decrease in absorbance with time was followed spectrophotometrically at 600 nm. Due to variable turbidity of the sample the cuvettes were placed as close as possible to the light source to keep resulting stray light to a minimum. The reaction was followed for 5 min and the initial rate of reaction calculated in terms of absorbance units $\times 10^{-5}$ per minute per $10^4$ cells. A calibration standard was run at the end of each set of samples by measuring the rate of reaction of a standard preparation of G6PD obtained from Sigma Chemical Co. (type XI activity 345 u $mg^{-1}$ protein).

### Results

*Cytochemical assay (6PGD)*

In the presence of 6PG and coenzyme the amount of NADP reduction is proportional to the 6PGD activity in the cell. With NBT present as a suitable final electron acceptor the resultant water-insoluble formazan particles precipitate in the cytoplasm of the cell. The formazan exhibits a strong affinity for protein (Heyden, 1979) and the reaction product is readily visible by light microscopy (Figure 1). The staining intensity is proportional to the activity of 6PGD in the cell. In the absence of either 6PG or NADP no staining occurred.

Approximately 100 individual cells were measured from each sample. In many of the normal smears the staining intensity of the majority of cells had low absorbance values. Examples of the data from a normal sample and one case of CIN and are illustrated in the frequency distribution histograms of the staining intensity (Figure 2). In Figure 2 no normal cells showed staining greater than 0.04 absorbance units. In contrast, many of the abnormal smears, i.e. CIN 1, 2 or 3 contained a proportion of cells with significantly higher absorbance values.

In order to compare the results from normal and precancerous samples certain absorbance values were chosen to represent 'cut off' points. The number of cells having absorbances higher than these values were counted for several samples. This number was then expressed as a percentage of the total number of cells counted in each sample. Figure 3 shows a summary of all the groups of data from normal and abnormal smears. The data comprise samples obtained from three different colposcopy clinics and represent the involvement of several research workers. Collection and analysis of these data covers a period of 3 years from 1987 to 1990; this has to be borne in mind when analysing the overlap between cytological and clinical diagnoses. In Figure 3 the 'cut-off' point chosen was 0.15 extinction units. The number of cells with staining intensities higher than 0.15 were counted and expressed as a percentage of the total number of cells measured. In the normal group only 12 out of 61 cases showed cells with staining intensities above 0.15 absorbance units, whereas 11 out of 14 cases of CIN 1, ten out of 11 cases of CIN 2 and ten out of 15 cases of CIN 3 exhibited staining intensities above O.D. 0.15. Although there is large overlap between grades there are more cells with high 6PGD activity in samples from patients with CIN 1, 2 and 3 compared to normal.

Some experiments were done using G6PD instead of 6PGD for cytochemical staining and the results were very similar in terms of differences between normal samples and CIN (data not included).

*Biochemical assay (G6PD)*

The activity of G6PD was monitored by following the rate of decrease in absorbance at 600 nm in the presence of lysed cells. This cycling procedure is analogous to the assay employed for cytochemical analysis in that reducing equivalents are passed to a final electron acceptor. Normal smears taken from patients visiting a routine health centre showed very low activity of G6PD using this method (Figure 4) as compared to cases of CIN. There is a definite correlation between G6PD activity and progressive stages of CIN. The total false negatives were 1 ($n = 33$; 3%) for CIN and the false positives were 3 ($n = 21$; 14%). For CIN 2 and CIN 3 there were no false negatives. Overlaps between grades of CIN are still present but there is a general trend towards higher activity with increasing grade of CIN. The two cases of invasive

**Figure 1** The presence of formazan deposits in cells from the squamous epithelium from the uterine cervix. Squamous cervix epithelial cells obtained after gradient separation and incubated with 2 mM 6-phosphogluconate, 0.5 mM NADP, 0.1% NBT and 0.14 mM PMS in 0.3 M glycl glycine buffer, pH 8.5 for 30 min at 37°C in the dark. Bar = 40 μm.

cancer in this study gave values of 6,000 and 10,000 OD units × $10^{-5}$ per minute per $10^4$ cells under the same conditions, and thus would be well off-scale in Figure 4.

For one sample of CIN 3 both cytochemical staining (6PGD) and the biochemical rate of reaction (G6PD) were studied. The results showed that 23% of epithelial cells had a staining intensity above 0.15 absorbance units; the equivalent biochemical activity was 90 O.D. units × $10^{-5}$ per minute per $10^4$ cells (see Figures 3 and 4). Table I summarises the false positives and false negatives for both cytochemical and biochemical methods of measurements based on a discriminatory line drawn at equivalent points in Figure 3 and 4.

### Discussion

The main objective of this study was to test the potential of biochemical measurements of the activity of glucose-6-phosphate dehydrogenase to discriminate between cervical intraepithelial neoplasias and normal tissue. In tackling this objective we have used our recently developed method (Slater *et al.*, 1992) for isolating purified suspensions of epithelial cells obtained from uterine cervix by normal sampling procedures. A secondary objective was to confirm that any increased pentose shunt activity, as measured biochemically with glucose-6-phosphate dehydrogenase (G6PD), was an

**Figure 2** Frequency distribution histograms of staining intensity from a normal and a pre-cancerous smear. Following Percoll gradient separation cells were stained for 6PGD as described in the text. Staining was measured by computerised microcytospectrophotometry at 540 nm. N = number of cells measured.

**Figure 3** Percentage of cells with staining intensity above extinction of 0.15 units at 540 nm. Cells were stained for 6PGD as described in the text and the intensity of staining was measured by quantitative microcytospectrophotometry. At least 100 cells were measured per sample. The figure shows the percentage of cells in each sampe that stained with an intensity greater than 0.15 units at 540 nm. Each point represents a different patient. An arbitrary discriminatory dashed line was drawn to produce the values for false negatives and positives given in the text.

expression of an increased activity in epithelial cells. For that purpose we have used quantitative microcytospectrophotometry to measure 6PGD in isolated individual cervical epithelial cells.

A stimulus for this study was the report (Rees *et al.*, 1970) more than 20 years ago, concerning G6PD activity in samples of vaginal fluid. These studies suffered from several inherent disadvantages including variability and limited extent of desquamation of cervical cells into the vaginal fluid, the subsequent undefined residence time in the vagina prior to analysis, and the presence of other cells such as erythrocytes, inflammatory cells and microorganisms that could contribute to measurements of total G6PD activity. The present results, which are much better than reported earlier by Rees *et al.* encourage us to proceed along this path to develop a simple and reliable biochemical screening test for CIN and cervical carcinoma.

G6PD is known to be increased in tumour cells generally (Weber, 1977) and in other dividing cells. Heyden (1974) has shown by histochemical techniques that G6PD is increased in lesions of the human uterine cervix. The enzyme is important not only for participating in the supply of pentose sugars for nucleic acid synthesis but also for producing NADPH and thus changing the redox couple $NADP^+$/NADPH. In this respect it is known that an increased emphasis in the reducing status of a cell can influence the up-regulation of thromboxane receptors on the cell surface. Thromboxane has been shown in a number of tumour systems to be a stimulator of cell division (Nigam & Averdunk, 1989). It is germane to note that changes in protein thiols have been reported in pre-malignant and malignant disorders of the uterine cervix (Schauenstein *et al.*, 1983; Bajardi *et al.*, 1983; Slater *et al.*, 1985; Nöhammer *et al.*, 1989; Benedetto *et al.*, 1990).

**Figure 4** G6PD activity in lysed cervix cells from normal patients and patients with CIN. The activity of G6PD, measured biochemically, in lysed samples of cervix epithelial cells were obtained and processed as described in the text. The figure shows the distribution of activity found in cells obtained from normal women, and from women with CIN-1, 2 or 3. Each point represents a different patient. An arbitrary discriminatory dashed line was drawn to produce the values for false negatives and positives given in the text.

**Table I** False negatives and false positives in normal samples and samples from CIN

| | *Normal false positives* | *CIN 1 false negatives* | *CIN 2 false negatives* | *CIN 3 false negatives* | *All CIN false negatives* |
|---|---|---|---|---|---|
| Cytochemistry | 4 (61) | 6 (14) | 1 (11) | 6 (15) | 13 (40) |
| | 6.6% | 43% | 9% | 40% | 33% |
| Biochemistry | 3 (21) | 1 (9) | 0 (10) | 0 (14) | 1 (33) |
| | 14% | 11% | 0% | 0% | 3% |

The data show the number of samples from normal women, and women with CIN 1, 2 or 3, that stained for 6PGD above or below the discriminatory line drawn in Figure 3 (cytochemistry). Similarly, the number of samples with activities of G6PD above or below the discriminatory line drawn in Figure 4 is shown (biochemistry). Numbers in parenthesis = total number of cells.

*Biochemical measurements*

As shown in Figure 4 there are significant differences in G6PD activity in lysed cell preparations obtained from samples identified as CIN 1, 2 or 3 compared with samples taken from normal females. With a discriminatory line drawn as indicated (equivalent to an activity of 17 O.D. units $\times 10^{-5}$ per minute per $10^4$ cells) there are no false negatives for samples of CIN 2 and 3 and approximately 10% for CIN 1. The false positive value for the normal group was approximately 14%.

The three 'unusually' high values for normals in Figure 4 could be due to (a) misdiagnosis, although all cases shown in Figure 4 had been examined cytologically; (b) as yet ill-defined influences such as concurrent hormone replacement therapy at the time of sampling, heavy smoking etc.

The one 'unusual' value for CIN 1 could again be due to misdiagnosis, or to failures in sampling and storage prior to collection for analysis.

The distribution of G6PD values in the normal group suggests that enzyme activity is not significantly affected by the stage of the menstrual cycle as the cases studied were randomly distributed through the cycle.

In contrast to the values for the normal group the values for CIN 1, 2 and 3 were widely distributed (Figure 4). This suggests that there may be different degrees of pre-malignancy within each of the conventional stages as suggested recently by Pinion *et al.* (1991). It is important to emphasise that the biochemical method, unlike the microcytospectrophotometry, uses only simple, inexpensive equipment and that it is easily adaptable for automation and routine use.

The use of measurements of G6PD activity in suspensions of cervical cells for diagnosis of CIN has been the subject of a provisional patent (no. 203633.4).

*Microcytospectrophotometric measurements*

The quantitative microcytospectrophotometry was used here to supplement the biochemical assays. It is clear that quantitative microcytospectrophotometry is entirely unsuited to routine use but in this study it has confirmed that the large differences found by biochemical assay of the pentose pathway (Figure 4) can be visualised and measured in individual cervical cells by microcytospectrophotometry (Figure 1). This was important to show in order to eliminate the possibility that the biochemical study was measuring G6PD in cells other than those of epithelial cells of the cervix. It should be noted that the microcytospectrophotometry entailed measuring formazan production in at least 100 cells per patient sample; the cytochemical results in Table I thus represent more than 10,000 individual measurements. The Table supports the differences found by biochemical measurements of pentose shunt activity between normal and cases of CIN. The false positives for normals in this case were comparable in both methods used. However, the false negatives for all the CIN groups were considerably worse for the cytochemistry than for the biochemistry. This suggests that the biochemical method used here is considerably more reliable. It measured at least $10^3$ times as many cells and is thus more sensitive in detecting small increases in enzyme activity. Overlap between grades would be expected, due to the uncertainty of direct correlation between the clinical classification of CIN as diagnosed by standard histology and biochemical changes as detected by our method. Many of the abnormalities observed in a PAP smear or by histology may not reflect the degree of biochemical changes occurring. For instance, the increased biochemical activity is not necessarily accompanied by enlarged nuclei (see Figure 1), indicating that these changes may be taking place long before any obvious morphological alteration. There may be therefore a genuine overlap between normal and CIN in terms of biochemistry. It is conceivable that some cases of CIN may be reverting back to normal and some normal cases may be in the process of transition to CIN. The spontaneous reversibility of CIN to normality has been shown previously in histopathology studies (Anderson, 1985; McIndoe *et al.*, 1984). This may explain some of the high values obtained for normal samples. It seems plausible to consider samples with G6PD activity greater than 17 O.D.

units $\times 10^{-5}$ per minute per $10^4$ cells as suspicious and warranting further investigation.

The cytochemical studies reported here confirm the conclusions reached above concerning the biochemical measurements: there is an increased 6PGD activity in cervix epithelial cells in CIN compared with normal. The values in CIN are widely dispersed (Figure 3), again suggesting that different degrees of biochemical aberration occur within these three morphologically defined sub-groups.

Our previous work on protein thiols in CIN and cervix carcinoma (Slater *et al.*, 1985; Nöhammer *et al.*, 1989; Benedetto *et al.*, 1990) has demonstrated the occurrence of a biochemical field effect in which biochemical changes occur in morphologically normal cells some distance from the morphologically identifiable lesion. An important recent finding (McDermott *et al.*, 1990) has found changes in G6PD in apparently normal areas of the breast distant from the site of a carcinoma. It will be of obvious importance to investigate whether a similar G6PD field effect occurs in CIN and cervix carcinoma; this is under active investigation.

This work has been generously supported by the Association of International Cancer Research.

We thank the staff of Hillingdon Hospital, in particular staff nurse Sandra Webster and the technical staff at the Istituto di Ginecologia e Ostetricia, Universita di Torino, Italy for their assistance in the supply of clinical samples.

We are grateful to Professor Patricia McLean for helpful comments.

### References

ANDERSON, M.C. (1985). The pathology of cervical cancer. *Cin. Obstet. Gynecol.*, **12**, 87–119.

BAJARDI, F., BENEDETTO, C., NÖHAMMER, G., SCHAUENSTEIN, E. & SLATER, T.F. (1983). Histophotometrical investigations on the content of protein and protein thiols in the epithelium and stroma of the uterine cervix. II. Intraepithelial neoplasia. *Histochemistry*, **78**, 95–100.

BENEDETTO, C., BAJARDI, F., GHIRINGHELLO,, B., MAROZIO, L., NÖHAMMER, G., PHITAKPRAIWAN, P., ROJANAPO, W., SCHAUENSTEIN, E. & SLATER, T.F. (1990). Quantitative measurements of the changes in protein thiols in cervical intraepithelial neoplasia and in carcinoma of the human uterine cervix provide evidence of a biochemical field effect. *Cancer Res.*, **50**, 6663–6667.

BLONK, D.I. (1969). Cytochemical examination of lactic dehydrogenase and glucose-6-phosphate dehydrogenase in smears from the uterine cervix. *Acta Cytol.*, **13**, 264–269.

BOARD, M., HUMM, S. & NEWSHOLME, E.A. (1990). Maximum activities of key enzymes of glycolisis, glutaminolysis, pentose phosphate pathway and tricarboxylic acid cycle in normal, neoplastic and suppressed cells. *Biochem. J.*, **265**, 503–509.

CANCER STATISTICS (1981). National Cancer Institute, Department of Medical Sciences, Ministry of Public Health, Bankok, Thailand.

CHAYEN, J., BITENSKY, L., AVES, E.K., JONES, G.R.N., SILCOX, A.A. & CUNNINGHAM, G.J. (1962). Histochemical demonstration of 6-phosphogluconate dehydrogenase in proliferating cells. *Nature*, **195**, 714–715.

COHEN, S. & WAY, S. (1966). Histochemical demonstration of pentose shunt activity in smears from the uterine cervix. *Br. Med. J.*, **i**, 88–89.

COULTON, L.A. (1977). Temporal relationship between glucose-6-phosphate dehydrogenase activity and DNA-synthesis. *Histochemistry*, **50**, 207–215.

ELLIOT, P.M., TUTTERSALL, M.H.N., COPPLESON, M., RUSSEL, P., WONG, F., COATES, A.S., SOLOMON, H.J., BANNATYNE, P.M., ATKINSON, K.H. & MURRAY, J.C. (1989). Changing character of cervical cancer in young woman. *Br. Med. J.*, **298**, 288–290.

GLOCK, G.E. & MCLEAN, P. (1954). Levels of enzymes of the direct oxidative pathway of carbohydrate metabolism in mammalian tissues and tumors. *Biochem. J.*, **56**, 171–175.

HEYDEN, H. (1974). Histochemical investigation of malignant cells. *Histochemistry*, **39**, 327–334.

HEYDEN, H. (1979). Enzymatic changes associated with malignancy with special reference to aberrant G-6-PD activity. In: *Quantitative Cytochemistry and its Application*, pp. 243–258. Pattison, J.R., Bitensky, L. & Chayen, J. (eds), Academic Press.

ISMAIL, S.M., COLCLOUGH, A.B., DINNEN, J.S., EAKINS, D.M.D., GRADWELL, E., O'SULLIVAN, J.P. & NEWCOMBE, R.G. (1989). Observer variation in histopathological diagnosis and grading of cervical intraepithelial neoplasia. *Br. Med. J.*, **298**, 707–710.

JOHNSON, D.B. & ROWLANDS, C.R. (1989). Diagnosis and treatment of cervical intraepithelial neoplasia in general practice. *Br. Med. J.*, **299**, 1083–1086.

LEE, J.A., SPARGO, D.J. & RILEY, P.A. (1991). Video absorbtiometry in diagnostic cytology: description of a new technique and a preliminary statistical evaluation. *J. Clin. Pathol.*, **44**, 749–752.

MCDERMOTT, E.W.M., BARRON, E.T., SMYTH, P.P.A. & O'HIGGINS, N.J. (1990). Premorphological metabolic changes in human breast carcinogenesis. *Br. J. Surg.*, **77**, 1179–1182.

MCINDOE, W.A., MCLEAN, M.R., JONES, R.W. & MULLINS, P.R. (1984). The invasive potential of carcinoma-*in situ*. *Obstet Gynecol.*, **64**, 451–458.

MISTRY, P., MERAZGA, Y., SPARGO, D.J., RILEY, P.A. & MCBRIEN, D.C.H. (1991). The effects of cisplatin on the concentration of protein thiols in the rat kidney. *Cancer Chemother. Pharmacol.*, **28**, 277–282.

MITCHELL, H., MEDLEY, G. & GILES, G. (1990). Cervical cancer diagnosed after negative results on cervical cytology: perspective in the 1980s. *Br. Med. J.*, **300**, 1622–1662.

MITCHELL, H. & MEDLEY, G. (1991). Longitudinal study of women with negative cervical smears according to endocervical status. *Br. Med. J.*, **337**, 265–267.

NIGAM, S. & AVERDUNK, R. (1989). Activation of arachidonate metabolism in rats after inoculation of tumor cells: preferential formation of Thromboxane $A_2$ and its proliferative effect on tumor cells. In: *New Trends in Lipid Mediators Research*, Braquet, P. (ed.), **3**, 319–324.

NILSON, B., ANDERSCH, B., HANSON, G. & HEYDEN, G. (1987). Histochemical investigation of cervical intraepithelial neoplasia. *Arch. Gynecol.*, **240**, 75–79.

NÖHAMMER, G., BAJARDI, F., BENEDETTO, C., KRESBACH, H., ROJANAPO, W., SCHAVENSTEIN, E. & SLATER, T.F. (1989). Histophotometric quantification of the field effect and the extended field effect of tumors. *Free Rad. Res. Comms.*, **7**, 129–137.

PINION, S.B., KENNEDY, J.H., MILLER, R.W. & MACLEAN, A.B. (1991). Oncogene expression in cervical intraepithelial neoplasia cancer of the cervix. *Lancet*, **337**, 819–820.

REES, K., SLATER, T.F., GIBBS, D.F. & STAGG, B.H. (1970). A modified assay for 6-phosphogluconate dehydrogenase in samples of vaginal fluid from women with and without gynecologic cancer. *Amer. J. Obstet. Gynec.*, **107**, 857–864.

SHIELD, P.W., DAUNTER, B. & WRIGHT, R.G. (1987). The PAP smear revisted. *Aust. NZ J. Obstet. Gynaecol.*, **27**, 269–282.

SCHAUENSTEIN, E., BAJARDI, F., BENEDETTO, C., NÖHAMMER, G. & SLATER, T.F. (1983). Histophotometrical investigations on the contents of protein and protein thiols of the epithelium and stroma of the human cervix. I. Cases with no apparent neoplastic alterations of the epithelium. *Histochemistry*, **77**, 465–472.

SLATER, T.F., BAJARDI, F., BENEDETTO, C., BUSSOLATI, G., CIANFANO, S., DIANZANI, M.U., GHIRINGHELLO, B., NÖHAMMER, G., ROJANAPO, W. & SCHAUENSTEIN, E. (1985). Protein thiols in normal and neoplastic human uterine cervix. *Febbs Lett.*, **187**, 267–271.

SLATER, T.F., BEESLEY, J., FLATMAN, A., HAMMOND, R. & JONAS, S.K. (1992). A method for preparing suspensions of human cervical epithelial cells that can be used for biochemical, cytochemical and cytological investigations. *Acta Cytologica*, (submitted).

SLATER, T.F. & SAWYER, B. (1962). A colorimetric method for estimating the pyridine nucleotide content of small amounts of animal tissue. *Nature*, **193**, 454–456.

SLATER, T.F., SAWYER, B. & STRÄULI, U. (1964). An assay procedure for nicotinamide-adenine dinucleotides in rat liver and other tissues. *Arch Internal. Physiol. Biochim.*, **72**, 427–447.

VAN NOORDEN, C.J.F. & TAS, J. (1980). Quantitative aspects of the cytochemical demonstration of glucose-6-phosphate dehydrogenase with tetranitro BT studied in a model system of polyacrylamide films. *Histochem. J.*, **12**, 669–685.

WEBER, G. (1983). Biochemical strategy of cancer cells and the design of chemotherapy: G.H.A. Memorial Lecture. *Cancer Res.*, **43**, 3466–3492.

WEBER, G. (1977). Enzymology of cancer cells. *New Engl. J. Med.*, **296**, 486–493.

WIDY, K. & KIERSKI, J. (1967). Succinic, lactic and glucose-6-phosphate dehydrogenases in pre-cancerous and cancerous stages in the uterine cervix. *Acta Cytol.*, **11,** 231–235.

WINKELSTEIN, W. Jr & SELVIN, S. (1989). Cervical cancer in young Americans. *Lancet*, **i,** 1385.

WOLFENDALE, M.R. (1991). Cervical samplers. *Br. Med. J.*, **302,** 1554–1555.

WOLFENDALE, M.R., KING, S. & USHERWOOD, M.M. (1983). Abnormal cervical smears: are we in for an epidemic? *Br. Med. J.*, **287,** 526–528.

WOOD, T. (1985). *The Pentose Phosphate Pathway*. Academic Press Inc: London.

YOSHIDA, A. (1986). *Glucose-6-phosphate dehydrogenase*. Yoshida, A. & Beutler, E. (eds). Academic Press Inc: London.