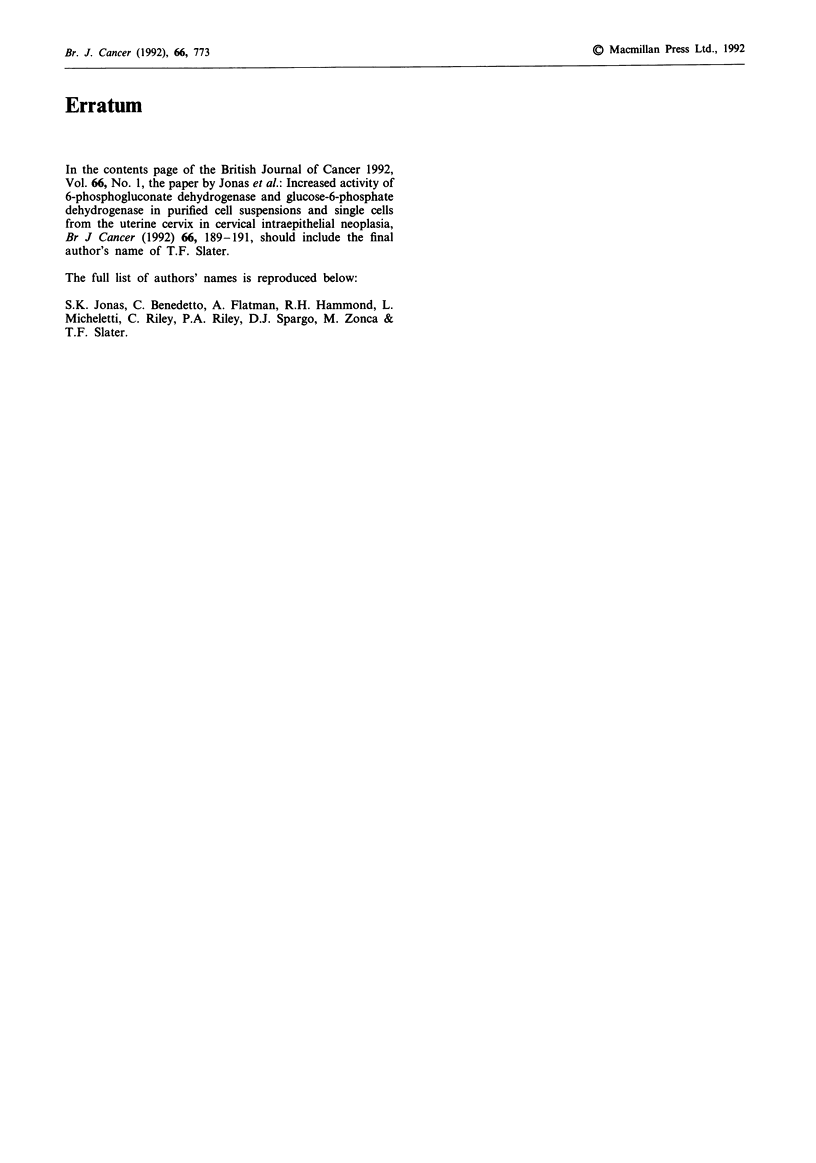# Erratum

**Published:** 1992-10

**Authors:** 


 © Macmillan Press Ltd., 1992

### Erratum

In the contents page of the British Journal of Cancer 1992, Vol. **66**, No. 1, the paper by Jonas *et al.*: Increased activity of 6-phosphogluconate dehydrogenase and glucose-6-phosphate dehydrogenase in purified cell suspensions and single cells from the uterine cervix in cervical intraepithelial neoplasia, *Br J Cancer* (1992) **66**, 189–191, should include the final author's name of T.F. Slater.

The full list of authors' names is reproduced below:

S.K. Jonas, C. Benedetto, A. Flatman, R.H. Hammond, L. Micheletti, C. Riley, P.A. Riley, D.J. Spargo, M. Zonca & T.F. Slater.