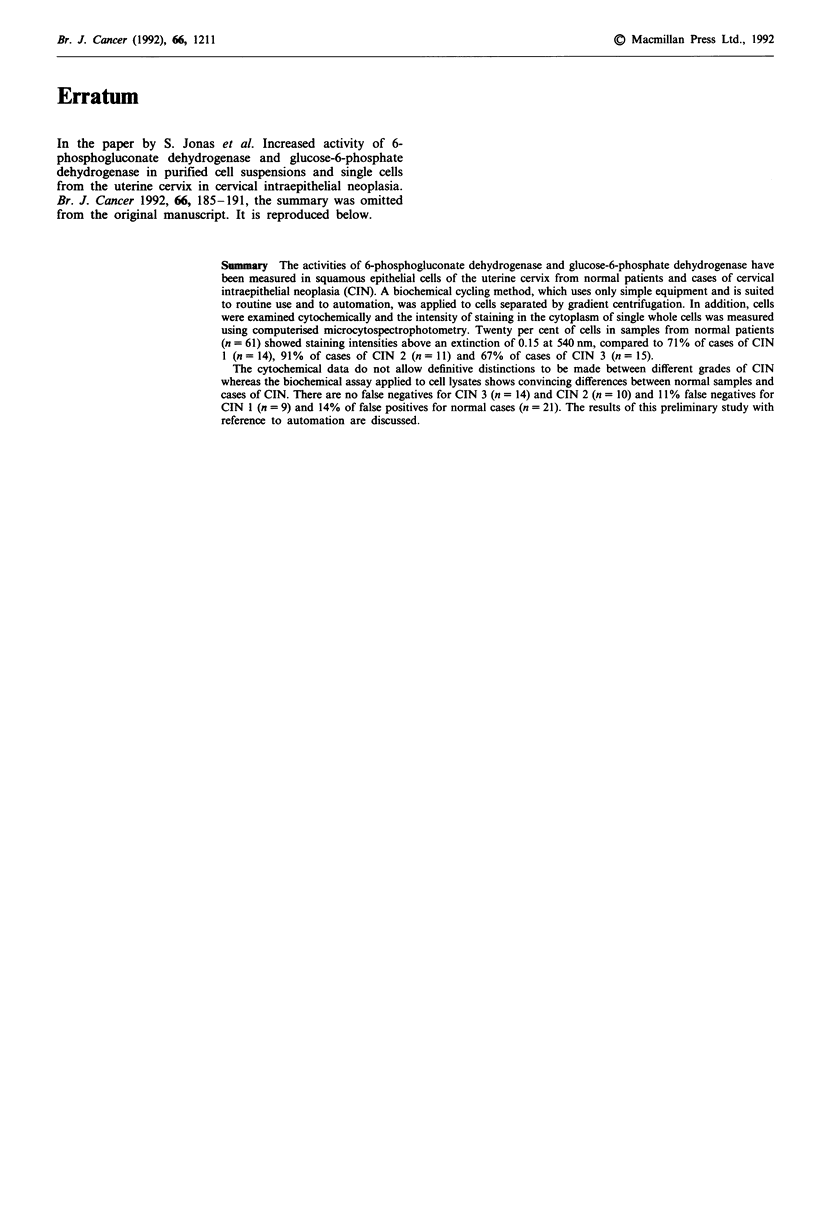# Erratum

**Published:** 1992-12

**Authors:** 

## Abstract

The activities of 6-phosphogluconate dehydrogenase and glucose-6-phosphate dehydrogenase have been measured in squamous epithelial cells of the uterine cervix from normal patients and cases of cervical intraepithelial neoplasia (CIN). A biochemical cycling method, which uses only simple equipment and is suited to routine use and to automation, was applied to cells separated by gradient centrifugation. In addition, cells were examined cytochemically and the intensity of staining in the cytoplasm of single whole cells was measured using computerised microcytospectrophotometry. Twenty per cent of cells in samples from normal patients (n = 61) showed staining intensities above an extinction of 0.15 at 540 nm, compared to 71% of cases of CIN 1 (n= 14), 91% of cases of CIN 2 (n= 11) and 67% of cases of CIN 3 (n= 15). The cytochemical data do not allow definitive distinctions to be made between different grades of CIN whereas the biochemical assay applied to cell lysates shows convincing differences between normal samples and cases of CIN. There are no false negatives for CIN 3 (n = 14) and CIN 2 (n = 10) and 11% false negatives for CIN 1 (n = 9) and 14% of false positives for normal cases (n = 21). The results of this preliminary study with reference to automation are discussed.


 © Macmillan Press Ltd., 1992

### Erratum

In the paper by S. Jonas *et al.* Increased activity of 6-phosphogluconate dehydrogenase and glucose-6-phosphate dehydrogenase in purified cell suspensions and single cells from the uterine cervix in cervical intraepithelial neoplasia. *Br. J. Cancer* 1992, **66**, 185–191, the summary was omitted from the original manuscript. It is reproduced below.

**Summary** The activities of 6-phosphogluconate dehydrogenase and glucose-6-phosphate dehydrogenase have been measured in squamous epithelial cells of the uterine cervix from normal patients and cases of cervical intraepithelial neoplasia (CIN). A biochemical cycling method, which uses only simple equipment and is suited to routine use and to automation, was applied to cells separated by gradient centrifugation. In addition, cells were examined cytochemically and the intensity of staining in the cytoplasm of single whole cells was measured using computerised microcytospectrophotometry. Twenty per cent of cells in samples from normal patients ($n = 61$) showed staining intensities above an extinction of 0.15 at 540 nm, compared to 71% of cases of CIN 1 ($n = 14$), 91% of cases of CIN 2 ($n = 11$) and 67% of cases of CIN 3 ($n = 15$).

The cytochemical data do not allow definitive distinctions to be made between different grades of CIN whereas the biochemical assay applied to cell lysates shows convincing differences between normal samples and cases of CIN. There are no false negatives for CIN 3 ($n = 14$) and CIN 2 ($n = 10$) and 11% false negatives for CIN 1 ($n = 9$) and 14% of false positives for normal cases ($n = 21$). The results of this preliminary study with reference to automation are discussed.